# Evaluation of exercise on individuals with dementia and their carers: a randomised controlled trial

**DOI:** 10.1186/1745-6215-11-53

**Published:** 2010-05-13

**Authors:** Arlinda Cerga-Pashoja, David Lowery, Rahul Bhattacharya, Mark Griffin, Steve Iliffe, James Lee, Claire Leonard, Sue Ricketts, Lyn Strother, Fiona Waters, Craig W Ritchie, James Warner

**Affiliations:** 1 Central & North West London NHS Foundation Trust, Greater London House, Hampstead Rd, London, NW1 7QY, UK; 2 Research Department of Primary Care & Population Health, University College London, Royal Free Campus, Rowland Hill St, London, NW3 2PF, UK; 3 Mile End Hospital, 3rd Floor Burdett House, Bancroft Road, London, E1 4DG, UK; 4 Independent - Not attached to any institution; 5 Avon and Wiltshire Mental Health Partnershp Trust, Older Peoples Mental Health Services, 53 Downs Way, Swindon, Witlshsire, SN3 6BW, UK; 6 Greater London Forum for Older People, 21 St Georges Road, London, SE1 6ES, UK; 7 Department of Psychological Medicine, Imperial College London, Claybrook Centre, 37 Claybrook Road, Hammersmith, London, W6 8LN, UK

## Abstract

**Background:**

Almost all of the 820,000 people in the UK with dementia will experience Behavioural and Psychological Symptoms of Dementia (BPSD). However, research has traditionally focused on treating cognitive symptoms, thus neglecting core clinical symptoms that often have a more profound impact on living with dementia. Recent evidence (Kales et al, 2007; Ballard et al, 2009) indicates that the popular approach to managing BPSD - prescription of anti-psychotic medication - can increase mortality and the risk of stroke in people with dementia as well as impair quality of life and accelerate cognitive decline. Consequently, there is a need to evaluate the impact that non-pharmacological interventions have on BPSD; we believe physical exercise is a particularly promising approach.

**Methods/Design:**

We will carry out a pragmatic, randomised, single-blind controlled trial to evaluate the effectiveness of exercise (planned walking) on the behavioural and psychological symptoms of individuals with dementia. We aim to recruit 146 people with dementia and their carers to be randomized into two groups; one will be trained in a structured, tailored walking programme, while the other will continue with treatment as usual. The primary outcome (BPSD) will be assessed with the Neuropsychiatric Inventory (NPI) along with relevant secondary outcomes at baseline, 6 and 12 weeks.

**Discussion:**

Designing this study has been challenging both ethically and methodologically. In particular to design an intervention that is simple, measurable, safe, non-invasive and enjoyable has been testing and has required a lot of thought. Throughout the design, we have attempted to balance methodological rigour with study feasibility. We will discuss the challenges that were faced and overcome in this paper.

**Trial Registration:**

ISRCTN01423159

## Background

"*Country air, moderate exercise, and a tonic regimen may retard the progress of senile dementia and suspend to some extent its termination*[1]". J.E.D. Esquirol, 1845

Dementia is a degenerative disease of the brain that leads to impairment of memory and global intellectual deterioration without affecting consciousness [2]. The prevalence rises sharply with increasing age: 5% of those over 65 and 20% for those aged over 80 will have dementia [3]. Dementia is also associated with a cluster of non-cognitive symptoms and behaviours that are an integral part of the syndrome. Commonly described as Behavioural and Psychological Symptoms of Dementia (BPSD), they include disturbed perception, thought content, mood or behaviour that frequently occur in patients with dementia [4]. Although recognised as core to the phenomenology of dementia in Alzheimer's seminal case studies [5], these symptoms have received relatively little attention from the research community. This is surprising given that upwards of 80% of people with dementia will experience BPSD at some point during the course of their illness [6]. Apart from the obvious distress for the person, BPSD are commonly associated with reduction in the quality of life for the person as well as their caregivers [7]; increase of caregiver stress [8], costs of care [9]; and unsurprisingly premature institutionalization [10].

Although there is some evidence supporting the treatment of BPSD with antipsychotics, there have been increasing concerns over the safety of these drugs for people with dementia. Two recent studies indicate that there is a long-term risk of mortality in patients with dementia who take antipsychotic medication [11,12]. Non pharmacological alternatives that have been reported to have a positive effects include music therapy, bright light therapy, behavioural interventions and exercise[13]. As well as the potential improvement in physical well being exercise has been demonstrated to have a positive effect on cognitive symptoms and mood [14,15]. Interventions such as exercise may offer a safer effective alternative to pharmacological treatments and could also be empowering to individuals with dementia and may reduce carers' burden.

Heyn et al carried out meta-analysis of exercise in dementia and reported data on 30 trials of exercise [16]. The authors reported on trials that included strength, cardiovascular or flexibility regimes; and analysed for functional, cognitive or behavioural outcomes. A significant positive effect of exercise on behavioural outcomes was reported (Effect Size = 0.54; 95% Confidence Interval = 0.36-.72). However these trials do not provide a full picture of the effectiveness of exercise on BPSD for a number of reasons. There was considerable heterogeneity in terms of the interventions, and exercise was often combined with other behavioural interventions. Thus, it is difficult to isolate the impact that exercise has had on behavioural outcomes. Some regimes were quite complex and require a high degree of physical fitness that would preclude many older adults with complex physical problems and moderate or profound dementia from performing them. Moreover, they were potentially unsustainable without the support of trained therapists. Finally, the relatively high cost of delivery and specialist input required may prevent the interventions being used more widely. Most trials included in the analysis were relatively small, with only two of the eight studies that reported effects on behaviours having samples in excess of 100 participants.

We have designed a study that aims to evaluate the effectiveness of exercise as a therapy for behavioural and psychological symptoms of dementia. This will be a pragmatic randomised trial conducted in a community cohort in the UK nested within a larger programme of research. The overarching programme investigates the impact of various psychosocial interventions for people with dementia, those who care for them and professionals that support them (Evidence Based Interventions in Dementia-EVIDEM). This has been a challenging design process, which has seen the project team - supported by an independent steering group - attempt to strike a balance between achieving a feasible and sustainable intervention, which could be widely adopted in the target population while maintaining scientific rigour in its evaluation. In the following, we describe the resultant and final methodology and discuss the benefits & limitations to our approach.

## Study hypothesis

A programme of tailored incremental exercise (through walking) improves the symptoms and outcome of Behavioural and Psychological Symptoms of Dementia

## Methods/Study Design

This trial has been granted ethical approval by the Outer North East London Research Ethics Committee, REC reference number: 09/H0701/67.

We will undertake a pragmatic, randomised, controlled, single-blind, parallel-group trial. Community-dwelling individuals with a diagnosis of dementia or suspected dementia who also have a carer willing and able to be a co-participant in the exercise regime will be recruited to this evaluation.

**Figure 1:**
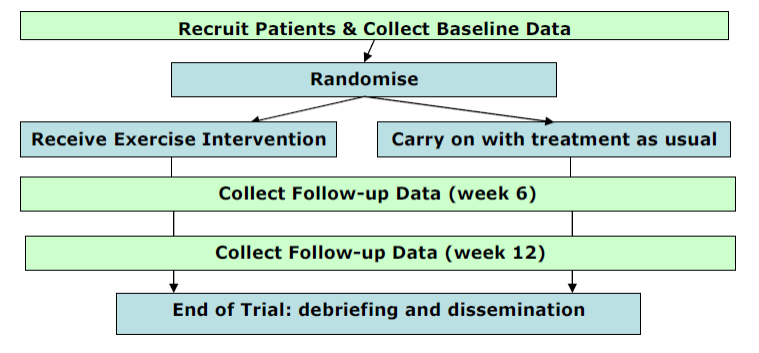
**Randomisation of participants and data collection**.

Participants will be randomly allocated to one of two arms: the treatment arm, which includes receipt of an individually tailored regime of walking (the intervention; exercise therapy-ET) in addition to treatment as usual (TAU) or the control arm which receives treatment as usual only (see Figure 1). Treatment as usual may include home care, attendance at day care facilities, visits by health professionals, receipt of medication, respite care etc.

Both the treatment and control arms will be re-assessed for all outcomes at weeks 6 and 12. Further telephone contact will occur at 26 weeks to assess adverse events, mortality, change in domiciliary status and adherence to exercise regime. The randomisation ratio for the two groups will be 1:1 (ET:TAU). An Independent randomisation officer will use a computer algorithm to perform the randomisation centrally after the initial interview, and will communicate the results to the participants, the GP and to the exercise therapist; but not to those collecting research data.

### Primary Objective

To determine the effectiveness of physical exercise delivered through a programme of incremental walking for treating behavioural and psychological symptoms of dementia (as defined by Neuropsychiatric Inventory scores) compared with treatment as usual (TAU).

### Secondary Objectives

To determine the effect of exercise on 1) the quality of life of people with dementia; 2) psychotropic medication usage; 3) move to a residential care facility; 4) mortality levels; 5) caregivers' perceived level of burden. We will also carry out an economic evaluation of the intervention and qualitatively explore participants' views about the intervention and barriers to treatment.

### Recruitment

The selection criteria for participation in the Evidem-E trial are outlined in Table 1 (Table 1). Participants with a clinical diagnosis of dementia or 'suspected dementia' with at least one significant BPSD symptom defined by the Neuropsychiatric Inventory (NPI)[17] (excluding the domains of delusions and hallucinations) will be eligible for the trial. Diagnosis of dementia will be confirmed in accordance with ICD-10 Diagnostic Criteria for Research (DCR-10). A risk assessment will be performed to assess the suitability of participants for the intervention at baseline. This assessment will include measurement for risk of falls through the Falls Risk Assessment Tool (FRAT) [18] and Timed Unsupported Steady Standing (TUSS) [19].

**Table 1:** Selection criteria for inclusion/exclusion from the Evidem-E trial

*Inclusion criteria*	*Exclusion criteria*
Diagnosis of: a. Dementia in primary or secondary care OR b. Suspected dementia confirmed by the researcher to ensure the DCR-10 criteria are met; Presence of a carer (professional, friend or family member, who does not necessarily have to live with the participant); Presence of BPSD measured through NPI. [NPI score in any one sub-set (except only hallucination or delusion) more than or equal to 2 in severity and more than or equal to 2 in frequency]; Consent of participant, or in the case of an individual who is not capable of giving informed consent, the assent of the participant with agreement of carer; Consent of carer.	Cardio-respiratory condition, neurological or musculo-skeletal condition of a degree that prevents participation to even the modified exercise regime unsafe or not possible. Three or more falls in the previous year ("frequent fallers") assessed by FRAT or high falls risk defined by TUSS; Uncontrolled medical problems, which the GP considers would exclude participants from undertaking the exercise programme; for example, acute systemic illness' such as pneumonia, poorly controlled angina, acute rheumatoid arthritis, unstable or acute heart failure; Sensory impairment to an extent that prevents dyad facilitated exercise; Participant or carer dissent to engage in the exercise programme.

Individuals with dementia known to the General Practitioner or secondary care will be identified and suspected new cases will be assessed to confirm diagnosis. Regular reminders about the study will be sent to participating practices and secondary care teams within the network catchment's area. A central register of all referrals will be maintained.

There are four potential sources of recruitment:

• The locally adopted Dementia Registry (DemReg) of people with dementia interested in participating in research.

• GP practices that are affiliated to the EVIDEM Research Group

• Memory assessment services and community mental health services (e.g. Admiral Nurses) recruited via CNWL NHS Foundation Trust

• Self referral

### Interventions

**Figure 2:**
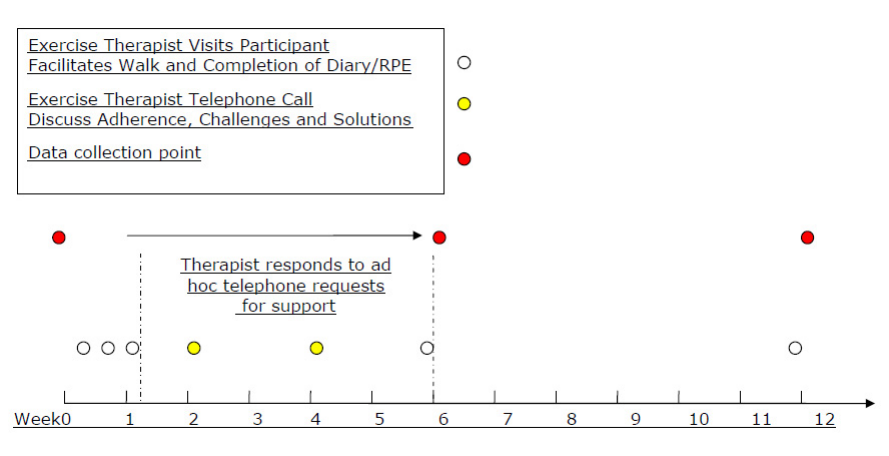
**Intervention schedule**.

Physical exercise will be delivered as an individually tailored regime of walking designed to become progressively intensive and last between 20-30 minutes. This will be facilitated by a qualified exercise therapist and delivered to participants in the treatment arm of the trial (Figure 2) in the area around their own home. The exercise therapist will facilitate physical exercise in the participant-carer dyad with the expectation that the dyad will perform the exercise regime regularly and independently of the therapist at least 5 times per week. The intervention has been structured so that the therapist adopts a phased withdrawal approach utilising both face-to-face contacts and telephone support contacts.

The dyad on the intervention group will also be asked to complete a visual analogue scale called the Rating of Perceived Exertion (RPE) [20]. Perceived exertion is based on the physical sensations a person experiences during physical activity, including increased heart rate, increased respiration or breathing rate, increased sweating, and muscle fatigue. Although this is a subjective measure, a person's exertion rating may provide a fairly good estimate of the actual heart rate during physical activity (Borg, 1998). The exercise therapist will not only assess the RPE but train participants to evaluate themselves and will encourage the dyad to extend walking exercise to 60-70% of RPE.

The participants in the treatment arm will be asked to record their daily activities and RPE throughout the 12 weeks of their participation using a diary designed to meet the specification of this study.

### Outcome Assessments

Assessments will be conducted according to Figure 1. Participants will be visited in either their own homes, or a mutually acceptable convenient location (e.g. GP's clinic), by a trained research worker (RW) masked to arm allocation.

The primary outcome measure will be changes to behavioural and psychological symptoms measured by the Neuropsychiatric Inventory (NPI) at baseline as well as 6 and 12 weeks of participation.

Secondary outcome measures will be changes to: participants' mental health (GHQ)[21], participants' quality of life (DemQOL-Proxy)[22], and caregivers' burden of caring (short ZBI) [23]. The secondary outcomes will also be measured at baseline, 6 and 12 weeks into the trial.

Participants' level of physical activity and compliance with the intervention will be measured through daily diaries, Rate of Perceived Exertion (RPE), blood pressure and heart rate monitoring. Diaries will also provide qualitative information in regards to participants' views about the intervention.

We will also carry out an Economic Evaluation through the Client Service Receipt Inventory [24], which will be administered at baseline and 12 weeks into the trial.

Domicile and mortality change will be measured at follow-up, 26 weeks into the trial via a structured telephone interview.

The exercise programme will be initiated and supervised by the Exercise Therapist. Baseline and subsequent evaluations will be undertaken by RW. An independent researcher (IR) will collect the primary outcome data (NPI) at weeks 6 and 12, which will be completed by telephone with the aim of minimising the risk of un-blinding group allocation to research workers.

### Sample size

Based on anticipated between-group absolute risk difference of 30% in number of people with BPSD symptoms measured by the NPI, a sample size of 146 participants will give 90% power to detect this difference with 95% confidence. This calculation includes a 20% attrition estimate. The sample size calculations have been described in Table 2.

**Table 2:** Power calculations

Control	Intervention	Difference	N (including compensation for 20% attrition)
0.8	0.4	0.4	70 (88)
0.8	0.5	0.3	116 (145)
0.8	0.6	0.2	238 (298)

(calculated by http://www.stat.ubc.ca/~rollin/stats/ssize/b2.html)

### Statistical Methods

Baseline demographic and outcome data will be compared between the two randomisation groups. Categorical data will be analysed using the chi-square test. Continuous data will be analysed using the t-test or if the data is found to be non-normally distributed, we will use the Wilcoxon Rank-Sum test.

Outcomes at 6 and 12 weeks will be compared between randomisation groups using analysis of co-variance (ANCOVA) for continuous data. The relevant baseline scores will be included as the co-variate in order to adjust for any potential baseline differences in the respective outcome. Categorical outcomes will be compared using the chi-square test.

All data will be analysed on an intention-to-treat basis, where appropriate imputation of missing data will be implemented. Under the assumption that data are missing at random multiple imputation will be used.

All analyses will be completed using the statistics package STATA.

## Discussion

Behavioural and psychological symptoms are an intrinsic feature of dementia that are often treated with drugs such as antipsychotics, which are potentially unsafe to use in this population. Alternative treatments for BPSD are required but there is a dearth of research in the area. We have tried to design a trial of exercise as therapy for BPSD. Designing such a trial provides challenges that drugs trials do not, such as: defining the treatment, maintaining blinding, defining clear measurable outcomes and ensuring the intervention is feasible and sustainable.

Exercise provides a promising intervention for BPSD, but further work is needed. Previous studies have reported the use of relatively complex exercise regimes including aerobic, strength, flexibility and balance training [25,26]. Most ageing individuals suffer from physical conditions such as cardio-respiratory illness, arthritis, and sensory and mobility impairments. It is important, therefore, that exercise therapy for this patient population is sensitive to this. Our study group and the independent Steering Group, which monitors and advises us, agreed that 'walking' is a feasible, sustainable and cost effective way of exercising. Walking is a simple way of exercising which enjoys all the benefits of more complex exercises but with the advantage of being sustained independently even by individuals with moderate to severe dementia and other physical conditions.

Careful consideration was taken in regards to blinding the researcher to the allocation status of the participants. The single blind design leaves us vulnerable to unmasking group allocation during the interaction between researcher and participant during visits. This everyday conversation is essential to building a rapport with participants and ensuring they feel comfortable in participating. All study personnel including researchers and therapists will repeatedly remind participants of the importance of not disclosing their group allocation. However, the risk will remain, and so we decided to include a second 'independent' researcher whom would collect our primary outcome data (NPI) at 6 and 12 weeks. The premise for this approach is that this researcher can take a less 'personal' tone and thus minimize the risk of conversation with the dyad that might un-blind group allocation. However, there remains a risk that the dyad will divulge information about the group they have been allocated to. Therefore the efficacy of blinding will be assessed at each time point, by personnel collecting data who will record which arm they believe each dyad is randomised to.

Separating the effect of psychosocial contact (from the exercise therapist) from the exercise intervention has proved to be the biggest challenge in our study. We decided to address this problem by introducing two periods during the intervention: 1-6 weeks whereby the participants in the intervention group will be trained and supported by an exercise therapist and 6-12 weeks when participants will receive absolutely no contact with the therapist. This stage will also help to assess the intervention's feasibility and sustainability in a community population. The symptoms of BPSD tend to be relatively stable over short time periods (i.e. months) so the two windows provided by this method should detect responses influenced purely because of exercise and not by any residual benefits of interaction with the therapist. Although we have tried to control for contacts between participants and the exercise therapist, we could not control for the social contact between the person with dementia and their carer. Dyadic walking may encourage not just physical activity but psychosocial support from the carer as well.

In terms of outcomes, we are using measures that are validated and widely used in research with individuals with dementia. We also gave considerable thought to the issue of measuring physical activity. Particular consideration was given to the use of equipment by the participants to record their individual activities e.g. Global Positioning System receivers and pedometers. The following issues were identified:

- Measurement accuracy and information bias (participants are likely to forget to wear their equipment or they may continue to wear equipment during non-activity time);

- Practicality and intrusion (wearing equipment for 12 weeks on daily basis can be intrusive and this may affect participation in the study and may give rise differential attrition);

- Methodological problems (introducing a self administered assessment of physical activity within the control group could constitute an intervention in its own right. There is potential for this to promote increased levels of physical activity within the control group and thus reduce the detectable effect size).

Therefore, we agreed to evaluate participants' level of physical activity through measurements of the participants' self reported Rate of Perceived Exertion (exercise group only). A proxy measure of fitness will be utilised to assess for compliance with intervention. Heart rate at rest will be assessed at the beginning, middle and end of the trial for all participants. We expect that should participants adhere to the prescribed exercise regime, their fitness will improve and ergo their heart rate at rest. We hypothesise that with such an increase in activity we may observe a reduction of BPSD. However we recognise that detecting such changes in cardiovascular health may be overambitious.

Assessing physical activity with minimum intrusion to study participants is challenging and we are relying on participants' self reports through daily dairies. Self-report is prone to information bias, especially when participants are not blinded to their group allocation. We may also encounter a significant Hawthorn effect whereby participation in the study and study hypothesis in particular may affect the behaviour of study participants. Thus, participants in the control group who believe they may benefit from regular exercise might increase their physical activity during the study period, which in turn may reduce any detectable effect due to the intervention.

Antipsychotic drugs, as discussed above, provide limited efficacy in treating BPSD as well as having established safety concerns. Exercise, on the other hand, may be effective but could also be associated with unknown risks. At present we can not discern whether the unconfirmed risk of the exercise is more substantial than the demonstrated risk of drugs. However, we have attempted to develop a design with robust risk event detection to mitigate this.

Designing the Evidem-E trial has proven to be very challenging but we have attempted to overcome the challenges without compromising scientific rigour or study feasibility. This trial is in accordance with the UK government's action plan to tackle the over-prescribing of antipsychotic drugs to people with dementia.

If this trial is successful, its findings may be applicable to a wide base of people diagnosed with dementia with minimal costs.

## Competing interests

The authors declare that they have no competing interests.

## Authors' contributions

JW conceived of the study, and along with DL, MG & RB developed the first draft of the protocol. AP wrote the first draft of the paper for publication. All authors significantly contributed to subsequent drafts and the final version submitted for ethical approval. All authors contributed to the design of the study, and have seen and approve of the final version. The quote from Esquirol was contributed by Dr Claire Hilton.

## References

[B1] JED Esquirol 'Mental Maladies: a treatise on insanity' fascimile of the English edition of 1845; published under the auspices of the Library of The New York Academy of Medicine New York: Hafner 1965

[B2] Rowley N Basic clinical science: describing a rose with a ruler 1991 London: Hodder and Stoughton

[B3] Livingston G Burns A, Levy R The scale of the problem Dementia 1994 1 London: Chapman and Hall 21 35

[B4] Finkel S Burns A Behavioral and Psychological Symptoms of Dementia (BPSD): a clinical and research update IPA Bulletin 1999 16 2

[B5] Alzheimer A Über eine eigenartige Erkrankung der Hirnrinde Allgemeine Zeitschrift fur Psychiatrie und Psychisch-gerichtliche Medizin 1907 64 146 48

[B6] Aalten P DeVugt ME Lousberg R Korten E Jaspers N Senden B Jolles J Verhey FRJ Behavioural Symptoms in Dementia: A Factor Analysis of the Neuropsychiatric Inventory; Dementia and Geriatric Cognitive Disorders 2003 15 99 105 12566599 10.1159/000067972

[B7] Colerick EJ George LK Predictors of institutionalization among caregivers of patients with Alzheimer's disease J Am Geriatr Soc 1986 34 492 498 10.1111/j.1532-5415.1986.tb04239.x 3722665

[B8] Finkel SI Costa e Silva J Cohen G Miller S Sartorius N Behavioural and psychological signs and symptoms of dementia: a consensus statement on current knowledge and implications for research and treatment Int Psychogeriatr 1996 8 Suppl 3 497 500 9154615 10.1017/s1041610297003943

[B9] Rabins PV Mace NL Lucas MJ The impact of dementia on the family JAMA 1982 248 333 335 10.1001/jama.248.3.333 7087127

[B10] Cohen-Mansfield J Assessment of disruptive behavior/agitation in the elderly: Function, methods and difficulties J Geriatr Psychiatry Neurol 1995 8 Suppl 1 52 60 7710649

[B11] Kales H Velenstein M Kim HM McCarthy JF Ganoczy D Cunningham F Blow FC Mortality risk in patients with dementia treated with antipsychotics versus other psychiatric medications American Journal of Psychiatry 2007 164 10 10.1176/appi.ajp.2007.06101710 17898349

[B12] Ballard C Hanney ML Theodoulou M Dougla S McShane R Kossakowski K Gill R Juszczak E Yu LM Jacoby R The dementia antipsychotic withdrawal trial (DART-AD): long term follow-up of a randomised placebo-controlled trial The Lancet 2009 8 10.1016/S1474-4422(08)70295-3 19138567

[B13] Devanand DP Lawlor BA Treatment of behavioural and psychological symptoms of dementia 2000 London: Martin Dunitz

[B14] Mather AS Effect of Exercise on Depressive Symptoms in Older Adults with poorly responsive depressive disorder: A randomised control trial The British Journal of Psychiatry 2002 180 411 415 10.1192/bjp.180.5.411 11983637

[B15] Williams CL Tappen RM Exercise training for depressed older adults with Alzheimer's disease Aging and Mental Health 2008 12 1 72 80 10.1080/13607860701529932 18297481 PMC2475651

[B16] Heyn P Abreu BC Ottenbacher KJ The effects of exercise training on elderly persons with cognitive impairment and dementia: a meta-analysis Archives of Physical Medical Rehabilitation 2004 85 1694 704 10.1016/j.apmr.2004.03.019 15468033

[B17] Cummings JL Mega M Gray K Rosenberg-Thompson S Carusi DA Gornbein J The Neuropsychiatric Inventory: comprehensive assessment of psychopathology in dementia Neurology 1994 44 12 2308 14 7991117 10.1212/wnl.44.12.2308

[B18] Nandy S Parsons S Cryer C Underwood M Rashbrook E Carter Y Eldridge S Close J Skelton D Taylor S Feder G Falls Prevention Pilot Steering Group Development and preliminary examination of the predictive validity of the Falls Risk Assessment Tool (FRAT) for use in primary care Journal of Public Health 2004 26 2 138 143(6) 10.1093/pubmed/fdh132 15284315

[B19] Studenski S Duncan PW Chandler J Samsa G Prescott B Hogue C Bearon LB Predicting falls: the role of mobility and nonphysical factors Journal of the American Geriatrics Society 1994 43 297 302 10.1111/j.1532-5415.1994.tb01755.x 8120315

[B20] Borg G Borg's Perceived Exertion and Pain Scales 1998 Human Kinetics Figure 7.3, page 49

[B21] Goldberg DP The detection of psychiatric illness by questionnaire 1972 London: Oxford University Press (Maudsley Monograph No 21)

[B22] Smith SC Lamping DL Banerjee S Harwood R Foley B Smith P Cook JC Murray J Prince M Levin E Mann A Knapp M Measurement of health-related quality of life for people with dementia: development of a new instrument (DEMQOL) and an evaluation of current methodology Health Technology Assessment 2005 9 10 10.3310/hta9100 15774233

[B23] Bédard M Molloy DW Squire L Dubois S Lever JA O'Donnell M The Zarit Burden Interview: a new short version and screening version Gerontologist 2001 41 5 652 7 11574710 10.1093/geront/41.5.652

[B24] Beecham J Knapp M Thornicroft G Costing psychiatric interventions Measuring Mental Health Needs 2001 2 London: Gaskell

[B25] Teri L Gibbons LE McCurry SM Logsdon RG Buchner DM Barlow WE Kukull WA LaCroix AZ McCormick W Larson EB Exercise plus behavioural management in patients with Alzheimer disease: A randomized controlled trial JAMA 2003 290 20015 2022 10.1001/jama.290.15.2015 14559955

[B26] Jirovec MM The impact of daily exercise on the mobility, balance and urine control of cognitively impaired nursing home residents International Journal of Nursing Studies 1991 28 145 151 10.1016/0020-7489(91)90004-M 1894462

